# Association between cannabis consumption and serum Klotho levels in middle-aged U.S. adults: NHANES cross-sectional analysis

**DOI:** 10.1186/s42238-025-00380-x

**Published:** 2026-01-06

**Authors:** Li Wang, Yanjing Ji, Tianxiao Wang, Weijian Wang, Gangjun Zong

**Affiliations:** 1 Department of Cardiology, Wuxi Clinical College of Anhui Medical University, Wuxi, 214000 Jiangsu China; 2 Department of Cardiology, The 904th Hospital of Joint Logistic Support Force of P.L.A., Wuxi, 214000 Jiangsu China

**Keywords:** Klotho Protein, Cannabis, National Health and Nutrition Examination Survey, Aging

## Abstract

**Background:**

With expanding U.S. cannabis legalization, clarifying its health impacts is critical. This study explores the association between cannabis use frequency and serum Klotho—a biomarker of aging and metabolic health.

**Methods:**

We analyzed 5,827 adults aged 40–59 years from National Health and Nutrition Examination Survey (NHANES) 2007–2016. Cannabis use was self-reported by Computer Assisted Self Interview (ACASI) system and validated questionnaires and serum Klotho concentrations were quantified by ELISA kits.

Weighted multivariable-adjusted generalized linear models evaluated associations, with subgroup analyses and interaction tests exploring potential effect modification by covariates. Sensitivity tests (restricted to non-smokers) were performed to investigate potential confounding by tobacco use. Additionally, E-value (assessing unmeasured confounding) was calculated to evaluate the robustness of primary findings. The population attributable fraction for Klotho reduction was calculated to quantify the population-level relevance of the observed association.

**Results:**

Cannabis use frequency demonstrated an inverse dose-dependent association with Klotho. Frequent users had significantly lower Klotho than never users (adjusted Estimate = -57.94, *P* = 0.027), with a significant trend (*P* for trend = 0.022). This association persisted in non-smokers (*P* = 0.047), confirming a cannabis-specific effect. E-value analysis showed the primary association was robust to unmeasured confounding (E-value = 4.77, 95% CI: 1.37, 8.57), exceeding the typical threshold for strong confounding (E-value > 1.5). Subgroup analyses revealed amplified Klotho reductions in frequent users with prolonged sedentary activity (> 240 min/day), higher vitamin D (serum 25(OH)D ≥ 72.5 nmol/L), and cancer (*P*-interaction < 0.05). The population attributable fraction was 3.13% (95%CI: 0.47%, 6.54%), indicating that approximately 3.1% of Klotho reduction in the study population was potentially attributable to frequent cannabis use.

**Conclusions:**

Frequent cannabis use is associated with reduced serum Klotho concentrations, a finding that may relate to biological aging-related mechanisms—particularly in metabolically healthier subgroups and individuals with prolonged sedentary behavior or cancer. This association remained robust after adjustment for numerous confounders and exclusion of smokers, highlighting the need for tailored monitoring of frequent cannabis users, especially vulnerable subgroups.

**Supplementary Information:**

The online version contains supplementary material available at 10.1186/s42238-025-00380-x.

## Introduction

The landscape of cannabis use in the United States has undergone significant changes over recent decades, particularly with the expanding legalization of cannabis for both medical and recreational purposes. The movement toward legalization gained momentum in the early 2000 s, culminating in Colorado and Washington becoming the first states to legalize recreational cannabis in 2012. Since then, the number of states permitting medical and/or recreational use has grown rapidly, reflecting shifting public attitudes. Recent studies highlight a notable rise in cannabis use across diverse demographics. For example, a national survey found that past-year cannabis use among U.S. adults increased significantly between 2013 and 2019, with nearly 10% of veterans reporting use during this period (Yockey & Hoopsick 2023). Similarly, cannabis use among young adults has risen steadily, with one study documenting an annual increase in prevalence from 2002 to 2018 (McCabe et al. 2021). The health implications of increased cannabis use remain complex. While some studies suggest therapeutic benefits for conditions such as chronic pain and anxiety (Page 2nd et al. 2020), others raise concerns about adverse effects on cardiovascular health and neurocognitive function (Page 2nd et al. 2020; Ren & Fishbein 2023).

Klotho, initially identified for its role in aging and longevity (Kuro-o et al. 1997; Abraham & Li 2022), acts as a multifunctional regulator across cardiovascular, renal, and neurological systems (Chen et al. 2021; Neyra et al. 2020; Sanz et al. 2021; Castner et al. 2023), and correlates with metabolic and inflammatory markers (Yamamoto et al. 2005; Abraham & Li 2022). Notably, cannabis use may interact with Klotho-relevant physiological pathways: cannabinoids can modulate inflammation, oxidative stress, and endocrine signaling—all of which are linked to Klotho regulation (Hajare et al. 2025). These potential mechanisms underscore the need to explore cannabis-Klotho associations, which have parallels to tobacco use in that greater smoking exposure correlates with reduced Klotho concentrations (Yao et al. 2022), while cessation is linked to Klotho recovery (Du et al. 2024).

By investigating cannabis-Klotho associations in the National Health and Nutrition Examination Survey (NHANES), this study addresses a novel research question with substantial public health relevance: as cannabis use becomes more widespread, understanding its impact on a key regulator of health and aging could inform clinical and policy decisions. Our analysis aims to clarify whether cannabis use, like tobacco, influences Klotho levels, and to identify potential dose-dependent or subgroup-specific effects—advancing knowledge at the intersection of substance use, molecular biology, and population health.

## Methods

### Study population

**Fig. 1:**
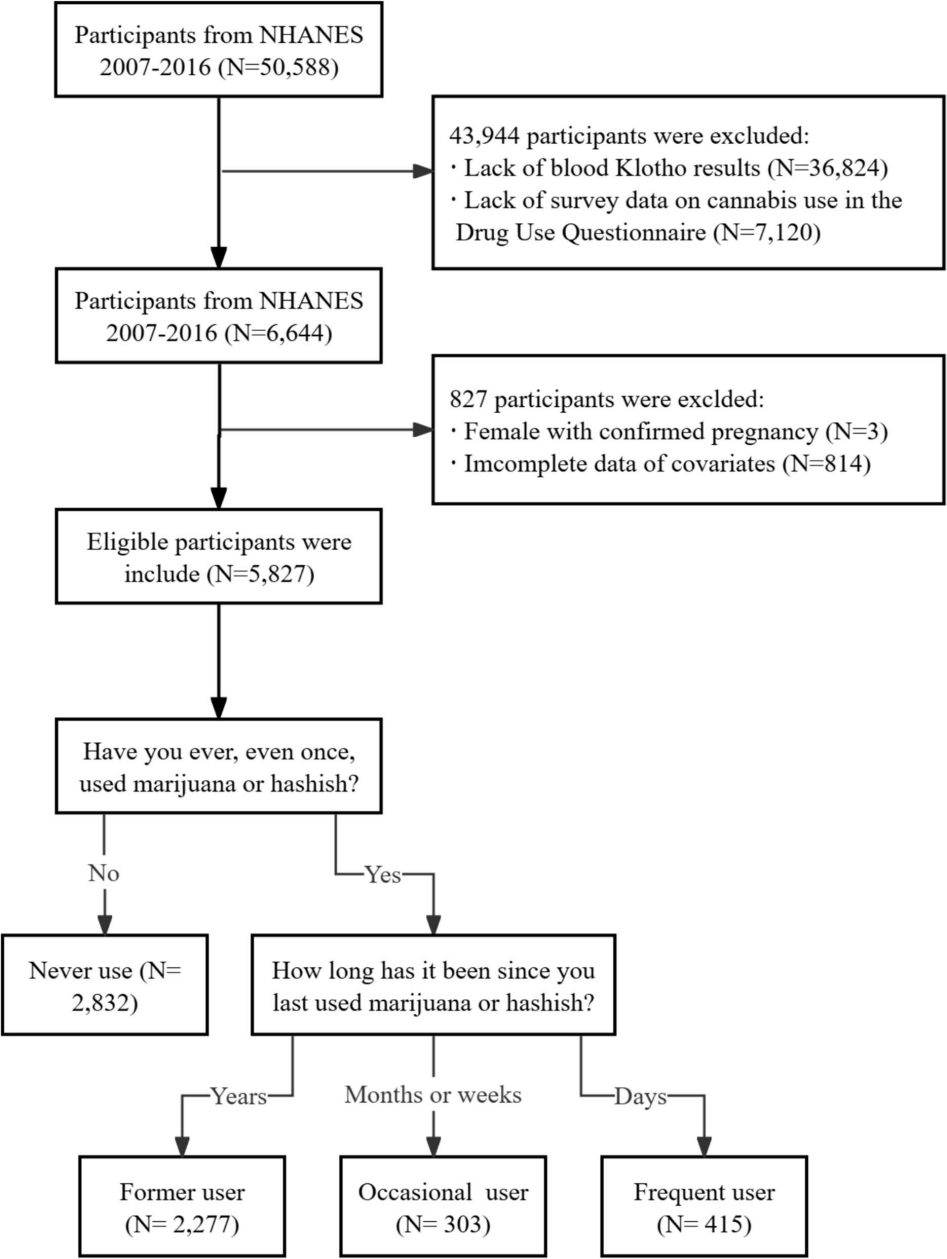
The flowchart of participant screening and grouping. Abbreviations: NHANES, National Health and Nutrition Examination Survey

This study analyzed data from five cycles (2007–2016) of the NHANES. Serum Klotho levels were measured in participants aged 40–79 years, while cannabis use data were collected for individuals aged 20–59 years. To align these age ranges, we focused on participants aged 40–59 years. From the initial sample of 50,588 individuals, we excluded 43,944 due to missing Klotho data (*N* = 36,824) or cannabis use survey responses (*N* = 7,120). Additionally, 3 pregnant individuals and 814 participants with incomplete covariate data were excluded (Fig. 1).

### Assessment of serum Klotho concentrations

Serum Klotho concentrations were quantified using a commercially available ELISA kit (IBL International, Japan). Duplicate analyses were averaged to ensure precision. Samples were stored at − 80 °C prior to processing at the Northwestern Lipid Metabolism and Diabetes Research Laboratory (University of Washington). The assay had a sensitivity of 4.33 pg/mL, with a mean serum Klotho concentration of 698.0 pg/mL. Detailed protocols are available on the NHANES website.

### Assessment of cannabis use

Cannabis use was self-reported via the NHANES Drug Use Questionnaire during the Mobile Examination Center (MEC) interview. Participants were asked: (1)“Have you ever, even once, used marijuana or hashish?”and (2)“How long has it been since you last used marijuana or hashish?”Respondents who answered “No” to the first question were classified as never use group (no lifetime cannabis use). Those who answered “Yes” were grouped as: (1) Former users: lifetime use, but no use in the past year. (2) Occasional users: lifetime use, with use in the past year but not recent (weeks to months ago). (3) Frequent users: lifetime use, with recent use (days ago) (Fig. 1).

### Other covariates

#### Demographic variables

Self-reported age, gender, race/ethnicity, education level, marital status, and family income-to-poverty ratio (PIR) were extracted from NHANES demographic files.

#### Weight-adjusted waist circumference index (WWI)

WWI is a novel central obesity assessment index, and prior studies suggest WWI inversely correlates with Klotho levels (Yin et al. 2024; Wen et al., 2025). WWI was calculated as waist circumference (cm) divided by the square root of body weight (kg):

$$WWI=WC\left(cm\right)/\sqrt{\text{Weight (kg)}}$$

The waist circumference (WC) and body weight measurements of participants were obtained by the physical examination.

#### Sleep duration and sedentary behavior

Sleep duration (hours/day) was derived from respondents self-reported sleep duration in the questionnaire. Sedentary time (minutes/day, excluding sleep) was based on the question “How much time do you usually spend sitting on a typical day (minutes)?” in the questionnaire.

#### Estimated glomerular filtration rate (eGFR) and Serum 25-hydroxyvitamin D

The serum creatinine of the participants was extracted from the laboratory data, eGFR of the participants was calculated based on the Chronic Kidney Disease Epidemiology Collaboration (CKD-EPI) equation (Killeen & Horowitz 2022), and the race correction was performed. Serum 25-hydroxyvitamin D of the participants was measured by a standardized liquid chromatography-tandem mass spectrometry method in the laboratory data.

#### Smoking status and alcohol consumption

Smoking status was self-reported via the Smoking–Cigarette/Tobacco Use questionnaire during in-person interviews. Participants were asked: (1) “Have you smoked at least 100 cigarettes in your entire life?” and (2) “Do you now smoke cigarettes?” Respondents who answered “No” to the first question were classified as nonsmokers. Those who answered “Yes” were grouped as: (1) Former smokers: Not currently smoking. (2) Occasional current smokers: Now smoke cigarettes some days. (3) Regular current smokers: Now smoke cigarettes every day. Alcohol consumption was assessed based on whether respondents consumed at least 12 cups of alcoholic beverages in the past 12 months.

#### Comorbidity prevalence

Hypertension was considered if one of the following conditions was met: (1) The participant was told by a doctor that they have hypertension. (2) The participant was taking antihypertensive medications. (3) The participant had elevated blood pressure (mean systolic blood pressure over 140 mmHg or diastolic blood pressure over 90 mmHg). Diabetes mellitus (DM) was considered if one of the following conditions was met: (1) The participant was told by a doctor that they have diabetes. (2) Glycohemoglobin HbA1c (%) was > 6.5. (3) Fasting glucose (mmol/L) was ≥ 7.0. (4) Random blood glucose (mmol/L) was ≥ 11.1. (5) 2-h oral glucose tolerance test (OGTT) blood glucose (mmol/L) was ≥ 11.1. (6) The participant was using antidiabetic agents. Cardiovascular diseases (CVD) was diagnosed if participants reported having congestive heart failure, coronary heart disease, heart attack, or angina. Chronic obstructive pulmonary disease (COPD) was defined as having any of the following: (1) Forced expiratory volume in 1 s (FEV1)/forced vital capacity (FVC) < 0.7. (2) Having been told by a doctor that they have emphysema or chronic bronchitis. Information regarding the presence of stroke and cancer were obtained from the questionnaire.

### Statistical analysis

Due to the complex sampling design of NHANES, we considered strata, primary sampling unit, and sampling weights in our data analysis according to the National Center for Health Statistics guidelines to estimate statistics for the entire US population. Statistical analysis was performed using R software (version 4.3.0, The R Foundation; https://www.r-project.org). Baseline characteristics were stratified by cannabis use frequency. The R package “survey” was used to calculate the frequency distribution of each variable ( UCLA 2025). Categorial variables were recorded by number count and percent, and continuous variables were expressed by mean ± standard deviation (SD). Normality assessment of continuous variables were evaluated by Q-Q plots and skewness/kurtosis indicators. Hypothesis verification for the linear regression models: (1) the residual normality hypothesis was verified by Q-Q plots and histograms; (2) the homoscedasticity hypothesis was verified by the scatter plot of residuals and fitted values. Univariate linear regression analysis was used to preliminarily explore association between baseline variables and serum Klotho concentrations. Multicollinearity among all covariates and cannabis use was assessed using multiple linear regression analysis. Variables exhibiting a variance inflation factor (VIF) exceeding 5 were excluded from subsequent model adjustments. Three multivariate linear regression models were conducted to evaluate the association between cannabis use and serum Klotho concentrations. Model 1 was adjusted for demographic variables. Model 2 was adjusted for demographic variables and comorbidity. Model 3 included all covariates identified without multicollinearity. Due to the similarity between cannabis and tobacco use in terms of behavior and health effects, we also performed comparative analysis with tobacco smoking and sensitivity analysis restricted to non-smokers. To assess the potential impact of unmeasured confounding on our primary finding, we calculated the E-value using the R package “EValue” (VanderWeele & Ding, 2017). To further quantify the population-level relevance of the observed association, we calculated the population attributable fraction (PAF) for Klotho reduction. Additionally, subgroup and interaction analyses were performed within the multivariable adjusted regression models. All analyses were considered significant if the *P*-value was less than 0.05.

## Results

### Baseline characteristics of participants

**Fig. 2:**
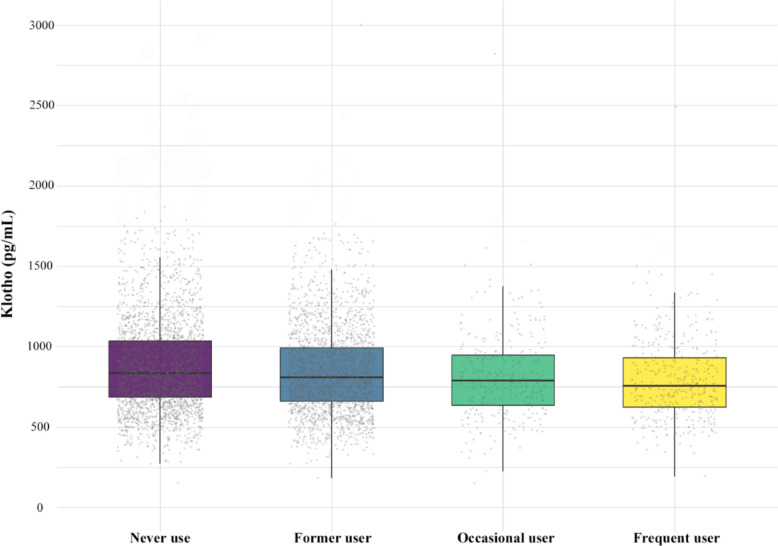
Boxplot of serum Klotho concentrations by cannabis use category. Each box represented the interquartile range (IQR, 25th to 75th percentile), the horizontal line within each box denotes the median, and whiskers extend to 1.5 × IQR. Points beyond the whiskers indicated outlier values. Data were presented as unadjusted values. Statistical comparisons between groups were performed using weighted linear regression, with never use cannabis as the reference category (*P* < 0.01)

As shown in Table 1, the survey-weighted characteristics of 5,827 participants were stratified by cannabis use frequency: never users (*n* = 2,832), former users (*n* = 2,277), occasional users (*n* = 303), and frequent users (*n* = 415). The boxplot displayed the distribution of serum Klotho levels in each group (Fig. 2). Frequent users had lower serum Klotho levels (803.25 pg/mL) than never users (881.40 pg/mL) and former users (853.71 pg/mL) (*P* < 0.01).

**Table 1:** Participant characteristics stratified by cannabis use frequency (*N* = 5,827)

Characteristic	N^a^	Overall *N* = 57,493,759^b^	Cannabis use frequency				*P *value
			Never use *N* = 22,936,984^b^	Former user *N* = 27,041,344^b^	Occasional user *N* = 3,346,875^b^	Frequent user *N* = 4,168,557^b^	
Gender (%)	5,827						<0.01
Female		3,028 (51%)	1,637 (58%)	1,090 (49%)	131 (42%)	170 (42%)	
Male		2,799 (49%)	1,195 (42%)	1,187 (51%)	172 (58%)	245 (58%)	
Age (years)	5,827	49.45 ± (5.65)	49.28 ± (5.74)	49.70 ± (5.54)	49.20 ± (5.79)	49.01 ± (5.66)	0.10
Race (%)							<0.01
Mexican American		950 (7.3%)	689 (13%)	203 (3.6%)	23 (3.2%)	35 (4.4%)	
Other Hispanic		615 (4.8%)	429 (8.2%)	148 (2.6%)	16 (2.2%)	22 (2.5%)	
Non-Hispanic White		2,578 (73%)	845 (60%)	1,330 (82%)	164 (77%)	239 (78%)	
Non-Hispanic Black		1,112 (9.3%)	474 (9.8%)	451 (7.9%)	82 (12%)	105 (12%)	
Other Race - Including Multi-Racial		572 (5.9%)	395 (8.8%)	145 (3.9%)	18 (5.7%)	14 (2.6%)	
Education level (%)	5,827						<0.01
Less than 9th grade		495 (4.1%)	402 (7.6%)	66 (1.6%)	11 (2.6%)	16 (2.2%)	
9-11th grade		811 (9.6%)	413 (10%)	272 (8.1%)	47 (10%)	79 (15%)	
High school graduate/GED or equivalent		1,262 (22%)	581 (22%)	481 (20%)	79 (25%)	121 (26%)	
Some college or AA degree		1,712 (31%)	694 (27%)	768 (33%)	101 (32%)	149 (38%)	
College graduate or above		1,547 (34%)	742 (33%)	690 (38%)	65 (30%)	50 (19%)	
Marital status (%)	5,827						
Married		3,554 (66%)	1,895 (72%)	1,369 (66%)	129 (51%)	161 (47%)	
Widowed		149 (2.2%)	72 (2.2%)	54 (2.0%)	15 (3.6%)	8 (2.0%)	
Divorced		883 (14%)	328 (10%)	401 (16%)	55 (18%)	99 (20%)	
Separated		264 (3.0%)	136 (3.1%)	86 (2.5%)	23 (4.3%)	19 (4.1%)	
Never married		608 (8.8%)	251 (8.0%)	245 (8.7%)	47 (11%)	65 (13%)	
Living with partner		369 (5.5%)	150 (4.2%)	122 (4.5%)	34 (12%)	63 (14%)	
PIR	5,827	3.38 ± (1.62)	3.21 ± (1.64)	3.64 ± (1.52)	3.35 ± (1.65)	2.64 ± (1.69)	<0.01
Sleep duration (hours)	5,827	6.91 ± (1.31)	6.96 ± (1.34)	6.87 ± (1.23)	6.91 ± (1.44)	6.89 ± (1.54)	0.27
Sedentary activity (minutes)	5,827	384.18 ± (206.50)	363.98 ± (204.92)	406.45 ± (208.24)	395.07 ± (197.84)	342.04 ± (193.13)	<0.01
WWI	5,827	11.00 ± (0.71)	11.05 ± (0.72)	10.96 ± (0.70)	10.96 ± (0.67)	10.97 ± (0.74)	0.01
Alcohol consumption (%)	5,827						<0.01
<12drinks/year		1,453(19%)	1,130 (35%)	266 (9%)	23 (4%)	34 (5%)	
≥12drinks/year		4,374 (81%)	1,702 (65%)	2,011 (91%)	280 (96%)	381 (95%)	
Smoking status (%)	5,827						
Nonsmoker		3,147 (55%)	2,036 (76%)	931 (44%)	95 (34%)	85 (22%)	
Former smoker		1,313 (24%)	415 (12%)	738 (34%)	63 (25%)	97 (29%)	
Occasional current smoker		210 (2.9%)	74 (2.0%)	83 (2.6%)	21 (6.8%)	32 (6.3%)	
Regular current smoker		1,157 (18%)	307 (9.4%)	525 (20%)	124 (34%)	201 (43%)	
Age started smoking cigarettes regularly (years)	2,680	17.05 ± (6.30)	17.51 ± (7.01)	16.87 ± (5.97)	17.67 ± (7.17)	16.70 ± (5.85)	0.31
Age when first tried cannabis (years)	2,995	17.64 ± (4.73)	-	17.75 ± (4.45)	18.39 ± (6.29)	16.30 ± (4.75)	<0.01
Last time used cannabis (days)	2,995	6489.06 ± (4800.73)	-	8275.24 ± (3841.43)	133.04 ± (413.95)	5.30 ± (23.83)	<0.01
Hypertension (%)	5,827						0.30
No		3,815 (68%)	1,896 (66%)	1459 (68%)	198 (72%)	262 (71%)	
Yes		2,012 (32%)	936 (34%)	818 (32%)	105 (28%)	153 (29%)	
Diabetes (%)	5,827						<0.01
No		4,993 (89%)	2,381 (86%)	1,977 (90%)	270 (93%)	365 (92%)	
Prediabetes		141 (2.3%)	70 (2.7%)	53 (2.2%)	9 (1.9%)	9 (1.3%)	
Yes		693 (8.9%)	381 (11%)	247 (7.7%)	24 (5.4%)	41 (7.0%)	
CVD (%)	5,827						0.10
No		5,548 (96.0%)	2,728 (96.8%)	2,152 (95.3%)	285 (96.6%)	383 (95.8%)	
Yes		279 (4.0%)	104 (3.2%)	125 (4.7%)	18 (3.4%)	32 (4.2%)	
Stroke (%)	5,827						0.17
No		5,706 (98.4%)	2,788 (98.7%)	2,229 (98.4%)	288 (97.2%)	401 (97.5%)	
Yes		121 (1.6%)	44 (1.3%)	48 (1.6%)	15 (2.8%)	14 (2.5%)	
COPD (%)	5,827						<0.01
No		5,443 (93.5%)	2,694 (95.0%)	2,105 (93.1%)	278 (93.1%)	366 (89%)	
Yes		384 (6.5%)	138 (5.0%)	172 (6.9%)	25 (6.9%)	49 (11%)	
Cancer (%)	5,827						<0.0
No		5,459 (91.8%)	2,692 (93.8%)	2,101 (90.5%)	282 (91.8%)	384 (89%)	
Yes		368 (8.2%)	140 (6.2%)	176 (9.5%)	21 (8.2%)	31 (11%)	
eGFR, mL/min/1.73m^2^	5,827	92.67 ± (15.62)	93.22 ± (16.09)	91.73 ± (15.23)	94.83 ± (14.82)	94.09 ± (15.76)	<0.01
Vitamin D (nmol/L)	5,827	70.63 ± (26.78)	67.42 ± (25.77)	73.15 ± (26.72)	70.75 ± (29.11)	71.77 ± (28.84)	<0.01
Klotho (pg/mL)	5,827	859.45 ± (298.00)	881.40 ± (294.28)	853.71 ± (291.26)	825.36 ± (330.20)	803.25 ± (322.91)	<0.01

^a^Unweighted N; ^b^N adjusted by NHANES weights (WTMEC2YR). Normality assessment of continuous variables were evaluated by Q-Q plots (Supplementary Figure S1-S11) and skewness/kurtosis indicators (Supplementary Table S1). The residual normality hypothesis was verified by Q-Q plots and histograms (Supplementary Figure S12). The homoscedasticity hypothesis was verified by the scatter plot of residuals and fitted values (Supplementary Figure S13). Weighted linear regression was used for intergroup comparison of continuous variables, and weighted chi-square tests with Rao-Scott correction were used for intergroup comparison of categorical variables

*Abbreviations*: *COPD* chronic obstructive pulmonary disease, *CVD* cardiovascular disease, *eGFR* estimated glomerular filtration rate, *PIR* family income-to-poverty ratio, *WWI* Weight-adjusted waist circumference index

Frequent cannabis users also exhibited distinct sociodemographic and behavioral characteristics compared with other groups (*P* < 0.01). Frequent users were more often male (58% vs. 42% never, 51% former), had lower family income-to-poverty ratios (2.64 vs. 3.21 never, 3.64 former), fewer college graduates (19% vs. 33% never, 38% former), and lower marriage rates (47% vs. 72% never, 66% former) (more divorced: 20%, cohabiting: 14%). Frequent users had higher alcohol consumption (95% with ≥ 12 drinks/year vs. 65% never, 91% former) and regular smoking (43% vs. 9.4% never, 20% former).

Additionally, frequent users had a higher prevalence of COPD (11%) and cancer (11%) compared to never users (5.0% and 6.2%, respectively), although the cancer association requires caution due to potential reverse causality or surveillance bias. No significant differences were observed in hypertension, CVD, stroke, or sleep duration across groups (*P* > 0.05). These findings indicated that frequent cannabis use was associated with a constellation of socioeconomic, behavioral, and health-related factors, including higher rates of substance use, lower socioeconomic indicators, and reduced levels of the anti-aging protein Klotho.

### Frequency-dependent associations between cannabis use and serum Klotho concentration

To preliminarily explore the association between covariates and the outcome, a univariate linear regression analysis of the potential influencing factors of the serum klotho concentration was shown in Supplementary Table S2. Gender, ethnicity, education level, WWI, sleep duration, alcohol consumption, smoking status, stroke and eGFR showed a significant association with serum Klotho concentration (*P* < 0.05). The VIF for all covariates remained below 5 (Supplementary Table S3), indicating no significant multicollinearity with cannabis exposure. Sixteen covariates were incorporated into weighted generalized linear models based on clinical relevance, univariate associations and statistical significance.

For binary cannabis use (Never use vs. Yes), in the unadjusted analysis, cannabis users exhibited a significantly lower Klotho concentration compared to never users (Estimate = −36.52, *P* < 0.001) (Table 2). This negative association remained significant after adjusting for demographic factors (Model 1: Estimate = −31.60, *P* = 0.001) and further for comorbidities (Model 2: Estimate = −31.27, *P* = 0.002) but lost significance when additional lifestyle and clinical covariates were included (Model 3: Estimate = −16.75, *P* = 0.117), indicating the impact of confounding factors (Table 2). For cannabis use frequency (Never use as the reference), univariate analysis revealed a stepwise decrease in Klotho concentration with increasing use frequency: former user (Estimate = −27.68, *P* = 0.005), occasional user (Estimate = −56.04, *P* = 0.013), and frequent user (Estimate = −78.15, *P* = 0.002), with a significant trend (*P* for trend < 0.05). In multivariate models, this dose–response pattern persisted after adjusting for demographic factors (Model 1, *P* for trend = 0.009) and comorbidities (Model 2, *P* for trend = 0.008). Even after comprehensive adjustment for lifestyle and additional clinical variables (Model 3), frequent user remained associated with lower Klotho concentration (Estimate = −57.94, *P* = 0.027), and the trend remained significant (*P* for trend = 0.022) (Table 2).

**Table 2:**
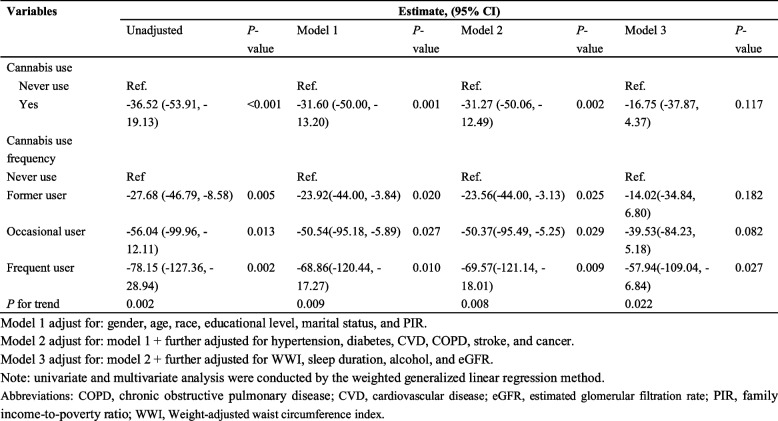
Univariate and multivariate analysis for the association between cannabis use and Klotho (*N* = 5,827)

### Comparative analysis with tobacco smoking and sensitivity to smoking confounding

Given behavioral and health effect similarities to cannabis use, we included smoking status as a positive control. As shown in Table 3, regular current smokers exhibited significantly lower serum Klotho concentrations across all models (*P* < 0.001 in unadjusted, Model 1, Model 2; *P* = 0.004 in Model 3), with a significant trend across smoking categories (*P* for trend < 0.001 in unadjusted, Model 1, Model 2; *P* = 0.001 in Model 3). In contrast, former smokers and occasional current smokers showed no significant associations with Klotho concentration in any adjusted model (*P* > 0.05). These findings confirm the expected negative association between regular cigarette smoking and serum Klotho, thereby validating the reliability of our analytical approach.

**Table 3:**
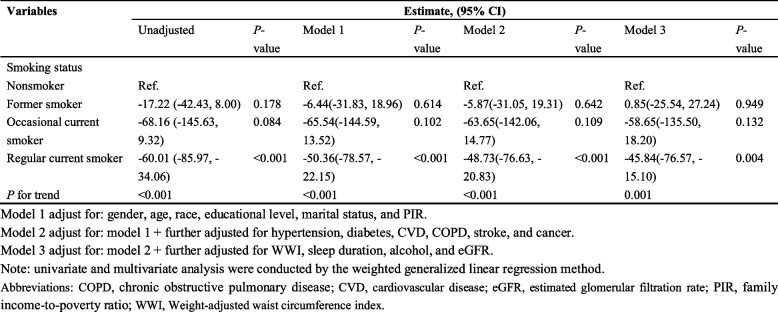
Univariate and multivariate analysis for the association between smoking status and Klotho (*N* = 5,827)

To further isolate the cannabis-specific effect and address potential confounding by smoking, we conducted a sensitivity analysis restricted to non-smokers (Table 4). In this subset, the dose–response relationship between cannabis use frequency and serum Klotho concentration remained robust. Frequent cannabis users exhibited significantly lower Klotho levels across all adjusted models: (Model 1: *P* = 0.009; Model 2: *P* = 0.011; Model 3: *P* = 0.047), with a consistent significant trend (*P* for trend < 0.001 in all models). In contrast, former and occasional cannabis users showed no significant associations with Klotho concentration in any adjusted model (*P* > 0.05). These findings confirm that despite potential co-occurrence of smoking and cannabis use, the association between frequent cannabis use and reduced serum Klotho is not confounded by tobacco smoking. Instead, it represents a distinct, cannabis-specific effect independent of smoking.

**Table 4:**
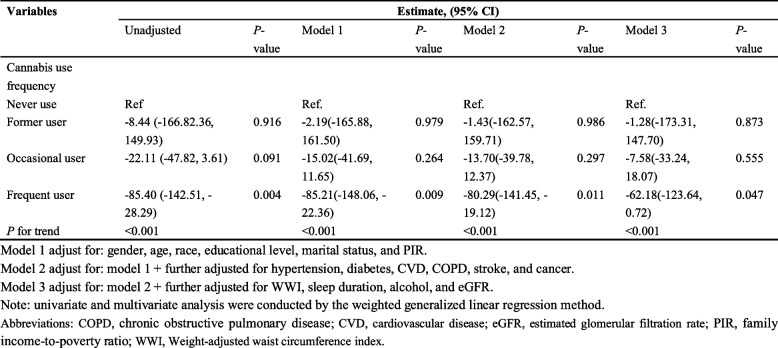
Univariate and multivariate analysis for the association between cannabis use and Klotho in non-smokers (*N* = 3,147)

### Evaluate the robustness of the association

To assess the potential impact of unmeasured confounding on our primary finding, we calculated the E-value manually. For the association between frequent cannabis use and reduced serum Klotho levels (adjusted β = −57.94, SE = 25.43), the E-value was 4.77 (95% CI: 1.37, 8.57). This suggests that an unmeasured confounder would need to be associated with both frequent cannabis use and serum Klotho levels by a factor of approximately 4.8 to fully explain the observed inverse association—exceeding the typical threshold for “strong unmeasured confounding” (E-value > 1.5) in observational studies (VanderWeele & Ding, 2017). Given that such a strong confounding effect is relatively uncommon in observational studies, our findings were robust to residual confounding, though we acknowledge that unmeasured biases cannot be completely ruled out.

### Subgroup and interaction analysis

**Fig. 3:**
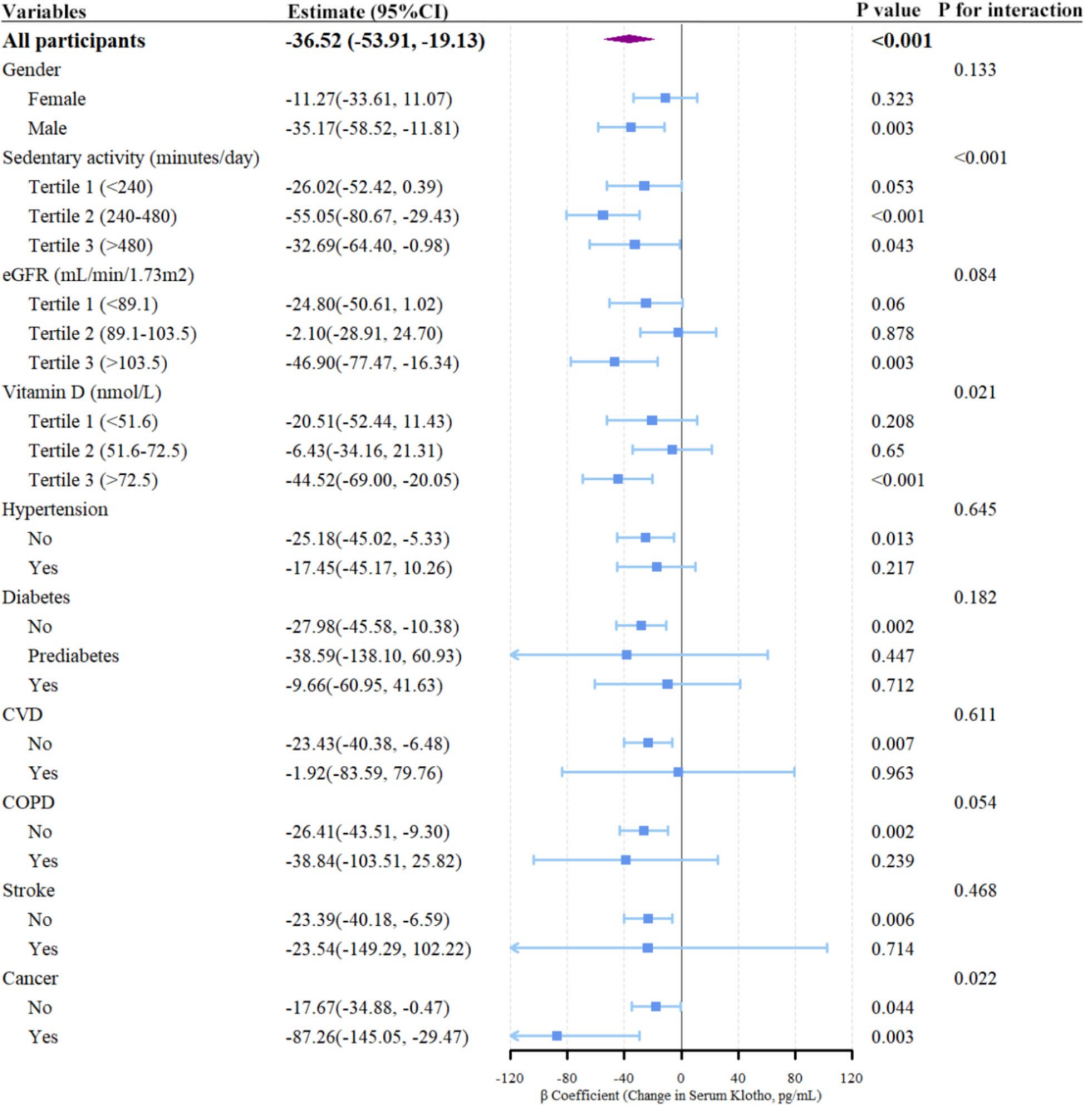
Forest plot of subgroup analyses for the association between cannabis use and serum Klotho. Reference group: never use cannabis. Values were β coefficients (95% CIs) representing the change in serum Klotho (pg/mL) in cannabis users relative to never users; Analyses were conducted using weighted generalized linear models adjusted for confounders in Model 3. Abbreviations: COPD, chronic obstructive pulmonary disease; CVD, cardiovascular disease; eGFR, estimated glomerular filtration rate

Subgroup analysis revealed significant differences in cannabis-related Klotho reductions across key demographics, lifestyle, and clinical subgroups (Fig. 3). For the demographic subgroup, the inverse association was significant in males (β = −35.17, *P* = 0.003) but not females (β = −11.27, *P* = 0.323; *P*-interaction = 0.133). For the lifestyle subgroup, a significant interaction (*P*-interaction < 0.001) was observed in sedentary activity. Klotho reductions were strongest in moderate (240–480 min/day: β = −55.05, *P* < 0.001) and high (> 480 min/day: β = −32.69, *P* = 0.043) sedentary subgroups, with a marginal association in the lowest tertile (< 240 min/day; *P* = 0.053). For the nutritional subgroup (Vitamin D), a significant interaction (*P*-interaction = 0.021) was observed and the association was only significant in the highest vitamin D tertile (≥ 72.5 nmol/L: β = −44.52, *P* < 0.001). A notable significant interaction (*P*-interaction = 0.022) was detected in cancer subgroup. Cannabis users with cancer had a marked Klotho reduction (β = −87.26, *P* = 0.003), whereas the association was weaker in those without cancer (β = −17.67, *P* = 0.044). Participants without comorbidities (renal insufficiency, hypertension, diabetes, CVD, COPD, and Stroke) had a significant reduction in Klotho (*P* < 0.05), while this significant association was not observed in participants with comorbidities (*P* > 0.05). No significant interactions were found for eGFR, hypertension, diabetes, or cardiovascular disease (all *P*-interaction > 0.05).

Sedentary activity, vitamin D, and cancer were identified as key modifiers of the cannabis-Klotho association by interaction analysis between covariates and cannabis use. The adverse effect of frequent cannabis use on Klotho was amplified in individuals with prolonged sedentary activity, higher vitamin D, and cancer. Based on these findings, the directed acyclic graph (DAG) was presented to visualize the assumed relationships between cannabis use, serum Klotho levels, and key confounders (Supplementary Figure S14).

### Population attributable fraction estimation

We defined "Klotho reduction" as serum levels below the study population median (817.7 pg/mL). Using adjusted relative risk (RR) from survey-weighted logistic regression (adjusted for 16 potential confounders) and the population prevalence of frequent cannabis use (Pe = 7.25%), we calculated the PAF as 3.13% (95%CI: 0.47%, 6.54%). This indicated that approximately 3.13% of Klotho reduction in the study population was attributable to frequent cannabis use. From a public health perspective, this finding suggested that targeted interventions to reduce frequent cannabis use could modestly mitigate the population burden of Klotho depletion, particularly in subgroups identified to be more vulnerable (e.g., cancer patients, individuals with prolonged sedentary behavior).

## Discussion

In this cross-sectional analysis of a nationally representative sample of U.S. adults aged 40 to 59 years (NHANES 2007–2016), we first reported a negative dose-dependent association between cannabis use frequency and Klotho, which was a specific effect independent of smoking behavior, although smoking had a similar effect on Klotho (Yao et al. 2022). Notably, this association exhibited pronounced effect modification in individuals with sedentary lifestyles, higher vitamin D, participants with cancer.

The growing societal normalization of cannabis underscores the urgency of clarifying its long-term health impacts, particularly on aging-related biomarkers. The Klotho protein, a pleiotropic regulator of aging pathways, exists in two functionally distinct isoforms: membrane-bound Klotho, which modulates fibroblast growth factor signaling, and soluble Klotho, a circulating form linked to antioxidative and anti-inflammatory properties (Kuro-o et al. 1997). Our findings align with emerging evidence of environmental influences on Klotho regulation. While Pedersen et al. reported no sex differences in serum Klotho concentrations across 120 healthy adults aged 19–66 using standardized immunoassays (Pedersen et al. 2013), Wang et al.’s NHANES-based analysis revealed significantly higher Klotho levels in females after adjusting for demographics, BMI, and tobacco exposure (Wang et al. 2024). This discrepancy may reflect methodological differences in population selection or confounder adjustment. Wang et al. further identified tobacco use and C-reactive protein (CRP) as male-specific Klotho suppressants, potentially attributable to males’ higher smoking prevalence and tobacco-associated chronic inflammatory states (Wang et al. 2024). Our findings revealed a similar sex dimorphism in Klotho levels among cannabis users. The amplified Klotho reduction in male cannabis users suggests shared biological pathways with tobacco toxicity, possibly involving androgen-mediated oxidative stress or inflammation.

Emerging evidence highlights bidirectional relationships between physical activity patterns and Klotho regulation (Vázquez-Lorente et al. 2024; Avin et al. 2014; Wu & Lu 2024; Hajare et al. 2025). In middle-aged sedentary adults, structured exercise interventions elevated plasma Klotho levels, suggesting activity-dependent Klotho upregulation (Vázquez-Lorente et al. 2024). Conversely, aging blunts this response: older adults (≥ 65 years) exhibit diminished Klotho increases after acute aerobic exercise compared to younger counterparts (25–45 years) (Avin et al. 2014). Notably, sedentary behavior modifies environmental influences on Klotho—a dose-dependent inverse association between caffeine intake and serum Klotho was exclusively observed in adults aged 40–79 with prolonged sedentariness (> 480 min/day; *P* for interaction < 0.05) (Wu & Lu 2024). We also found a correlation between lifestyle and Klotho levels in sedentary populations. In adults aged 40–59 who were long-term sedentary (> 240 min/day), we observed a dose-dependent negative correlation between cannabis use frequency and serum Klotho levels.

Klotho serves as a pivotal regulator of kidney homeostasis through its modulation of fibroblast growth factor 23 (FGF-23) signaling, vitamin D metabolism, and phosphate balance (Haussler et al. 2012). Vitamin D upregulates Klotho expression, while Klotho itself exerts negative feedback control over vitamin D synthesis (Haussler et al. 2012), forming a critical endocrine axis for calcium-phosphate homeostasis. Dysregulation of this axis elevates cardiovascular disease risk and mortality (de Borst et al. 2011; John et al. 2011). Our interaction analysis indicated that vitamin D status acted as an effect modifier of the association between cannabis use and serum Klotho levels. Lower vitamin D levels were typically associated with lower Klotho levels. Due to the ceiling effect, the decline in Klotho among individuals in lower vitamin D tertiles was less significant, whereas the decline in Klotho was more significant among those in the highest vitamin D tertile.

Klotho’s dual role as an aging suppressor and nephroprotective agent is well-established (Hajare et al. 2025). Epidemiologic studies demonstrate positive correlations between serum Klotho and eGFR, particularly in high-risk populations (elderly, obese, or diabetic individuals), while preclinical models link Klotho deficiency to oxidative stress, inflammation, and accelerated chronic kidney disease (CKD) progression (Zhang et al. 2022; Shikida et al. 2021). We found that frequent cannabis users in the highest eGFR tertile exhibited profound Klotho depletion (β = −46.9, *P* = 0.003). This results can be partially explained by the ceiling effect—the individuals with chronic diseases already have low Klotho levels, so further reduction due to cannabis use is limited. Given Klotho’s capacity to mitigate oxidative damage and fibrotic pathways (Yamamoto et al. 2005), its decline could create a vicious cycle—reduced Klotho accelerates renal injury, while kidney dysfunction further suppresses Klotho synthesis (Shikida et al. 2021). Clinically, serial Klotho monitoring in cannabis users may enable early identification of subclinical nephron stress preceding eGFR decline.

In addition to cancer, individuals without comorbidities had a greater reduction in Klotho from cannabis use compared to those with comorbidities, which was consistent with the subgroup analysis results of eGFR. Even though these results can be partly explained by the ceiling effect, it is still advisable for healthy individuals to minimize the use of recreational cannabis to mitigate potential risks. Similarly, for male and people with sedentary behavior, it is necessary to strengthen the publicity and education on the hazards of tobacco use such as cigarettes and cannabis, reduce the incidence of chronic diseases and malignant tumors, reduce passive smoking and cross-generational health effects, and finally reduce social medical expenses and social disease burden, which can achieve double improvement of health benefits and economic efficiency.

### Limitations

Several limitations of the present study should be acknowledged, which need to be considered when interpreting the findings.

First, the cross-sectional design of NHANES precludes definitive causal inference between frequent cannabis use and serum Klotho depletion. We observed an inverse association, but cannot rule out reverse causality. Longitudinal studies with repeated assessments of cannabis use and serial Klotho measurements are therefore needed to establish temporal order and potential causality.

Second, our study population was restricted to adults aged 40–59 years, a limitation dictated by the eligibility criteria for both the NHANES cannabis use questionnaire (which targets specific age ranges across survey cycles) and serum Klotho measurement in the 2007–2016 dataset. This age restriction limits the generalizability of our findings to individuals younger than 40 or older than 59 years, as age-related differences in Klotho metabolism (e.g., naturally declining Klotho with advanced age) and cannabis use patterns (e.g., higher prevalence in younger adults) may alter the observed association in other age groups.

Third, the granularity of cannabis exposure data was limited. The NHANES questionnaire captures cannabis use frequency (never/former/occasional/frequent) but lacks details on critical exposure metrics: including the tetrahydrocannabinol (THC) and cannabidiol (CBD) content of consumed cannabis, duration of regular use (e.g., years of frequent use), and consumption intensity (e.g., grams per use or frequency of use per week). These unmeasured factors hinder precise characterization of the dose–response relationship, making it difficult to determine whether specific cannabis components or use patterns drive Klotho reductions.

Fourth, residual confounding cannot be fully excluded. While we adjusted for a comprehensive set of covariates (demographics, comorbidities, lifestyle factors like sedentary activity and smoking), unmeasured or imprecisely measured variables may contribute to the observed association. Additionally, potential measurement error in self-reported cannabis use (e.g., underreporting due to stigma around cannabis use, even in legalized contexts) may introduce misclassification bias, though the use of NHANES’ validated questionnaire mitigates this to some extent.

## Conclusion

Our study provided the first epidemiological evidence that frequent cannabis use was associated with reduced serum Klotho concentrations in middle-aged adults (40–59 years)—with a magnitude comparable to regular tobacco smoking—this association was dose-dependent and cannabis-specific. Notably, metabolically healthier subgroups and individuals with prolonged sedentary behavior or cancer were disproportionately vulnerable to this effect. Amid expanding U.S. cannabis legalization, these findings underscored the need for subgroup-targeted public health risk assessment and tailored Klotho monitoring in clinical practice for vulnerable frequent users. Future research should focus on longitudinal studies, mechanistic investigations, and granular cannabis exposure data to refine dose–response relationships.

## Supplementary Information

Supplementary Material 1.

Supplementary Material 2.

## Data Availability

The datasets generated and/or analyzed during the current study are available in the NHANES data portal (https://wwwn.cdc.gov/nchs/nhanes/analyticguidelines.aspx).
