# Supplementary material for: Association between cannabis consumption and serum Klotho levels in middle-aged U.S. adults: NHANES cross-sectional analysis
Source: J Cannabis Res. 2026 Jan 6;8:18. doi: 10.1186/s42238-025-00380-x (PMC12870139; doi:10.1186/s42238-025-00380-x)
Supplement: Supplementary file 1 — Supplementary Material 1. [file 42238_2025_380_MOESM1_ESM.pdf]

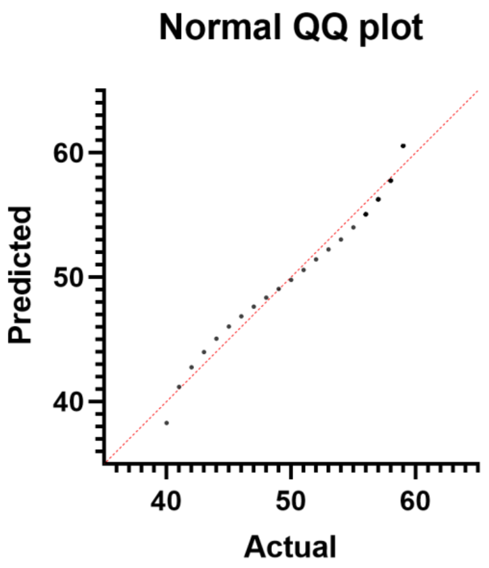

Figure S1 Normal QQ plot of age

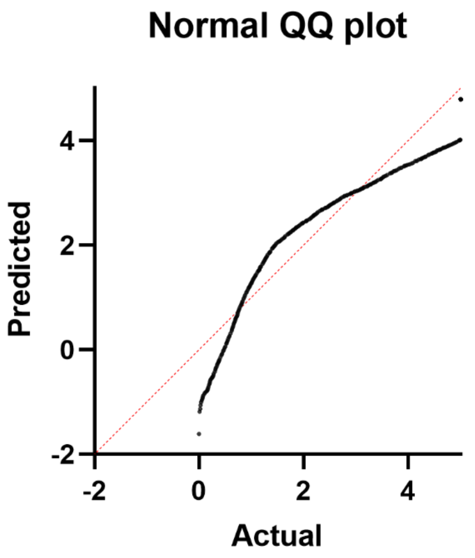

Figure S2 Normal QQ plot of family income-to-poverty ratio (PIR)

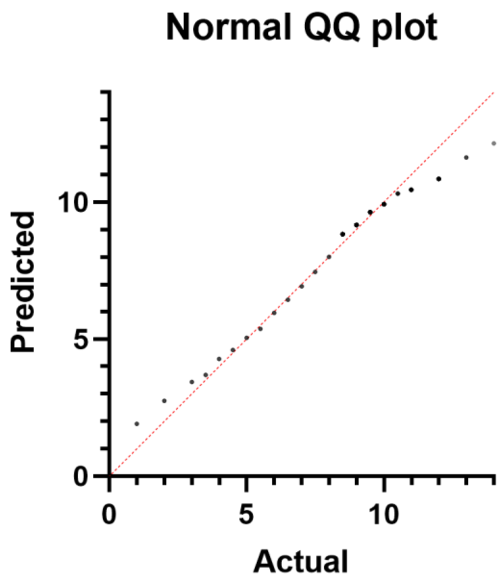

Figure S3 Normal QQ plot of sleep duration

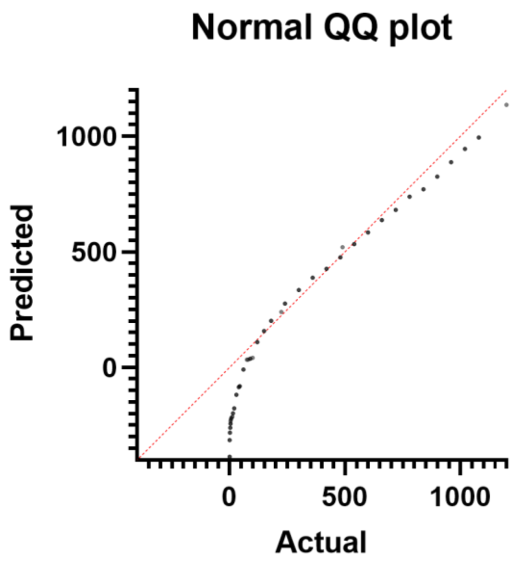

Figure S4 Normal QQ plot of sedentary activity

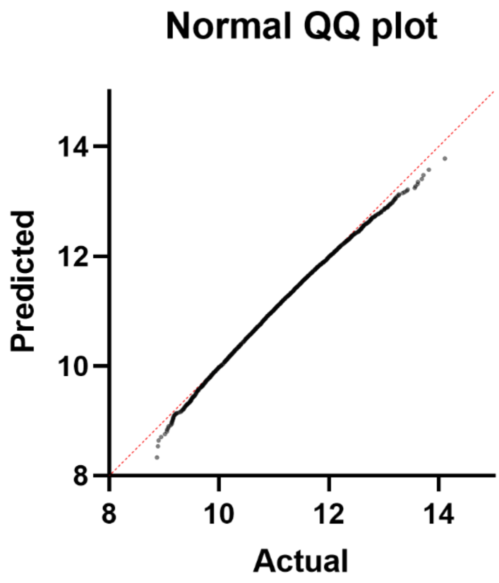

Figure S5 Normal QQ plot of Weight-adjusted waist circumference index (WWI)

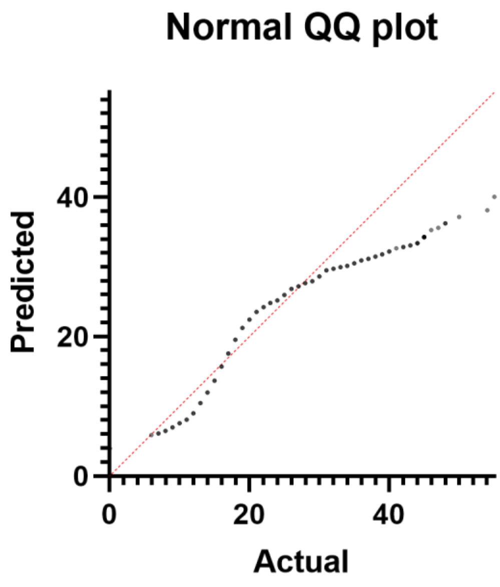

Figure S6 Normal QQ plot of variable (age started smoking cigarettes regularly)

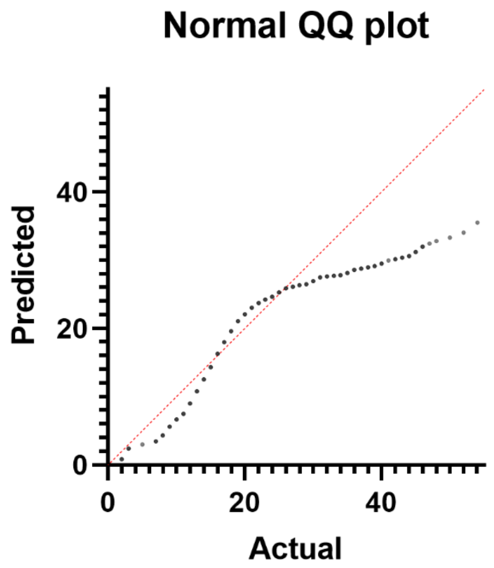

Figure S7 Normal QQ plot of variable (age when first tried cannabis)

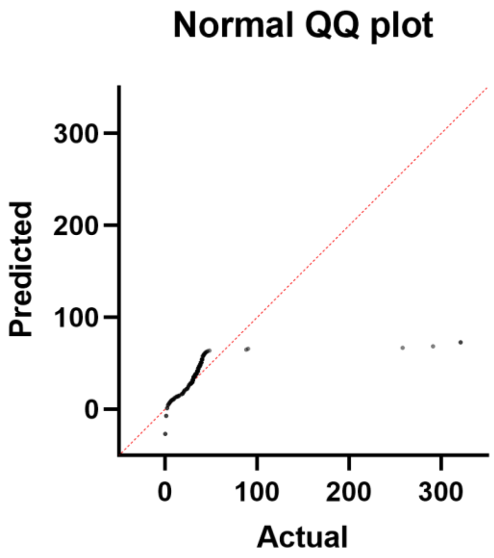

Figure S8 Normal QQ plot of variable (last time used cannabis)

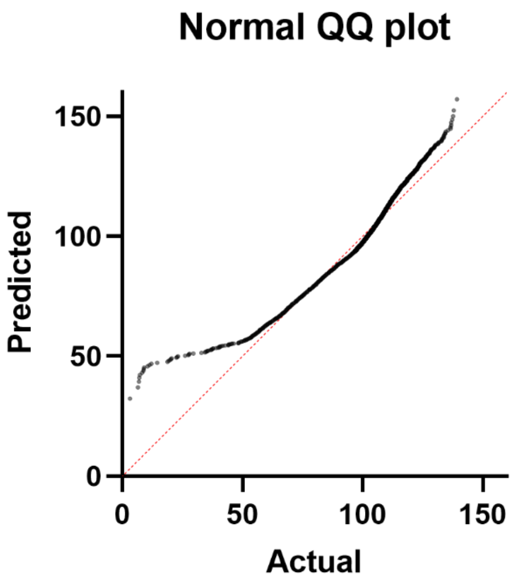

Figure S9 Normal QQ plot of estimated glomerular filtration rate (eGFR)

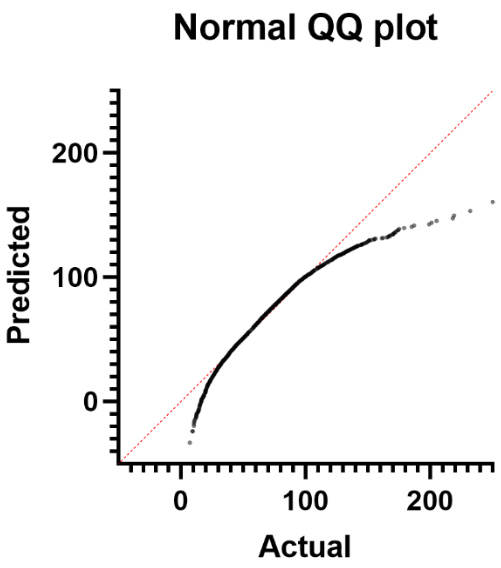

Figure S10 Normal QQ plot of Vitamin D

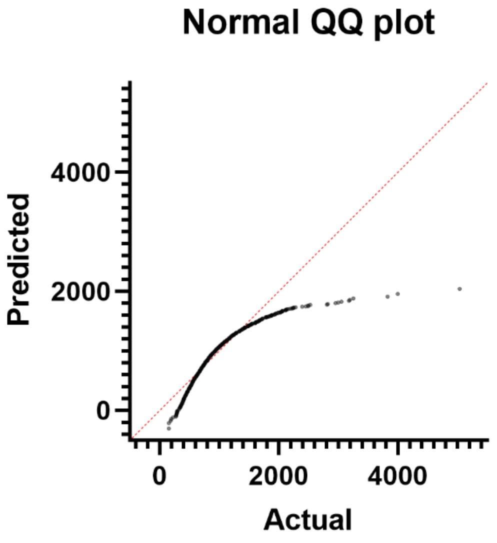

Figure S11 Normal QQ plot of Klotho

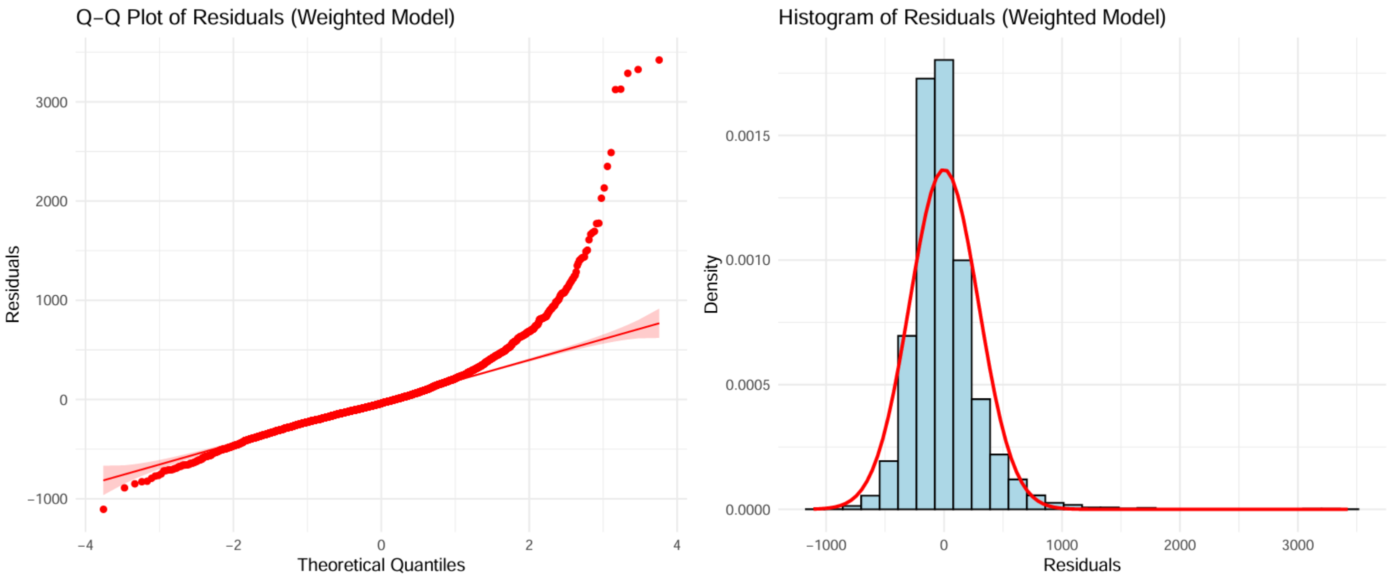

Figure S12 Normality of residuals for continuous independent variables. Q-Q plots and histograms showed

that the residuals were approximately normally distributed.

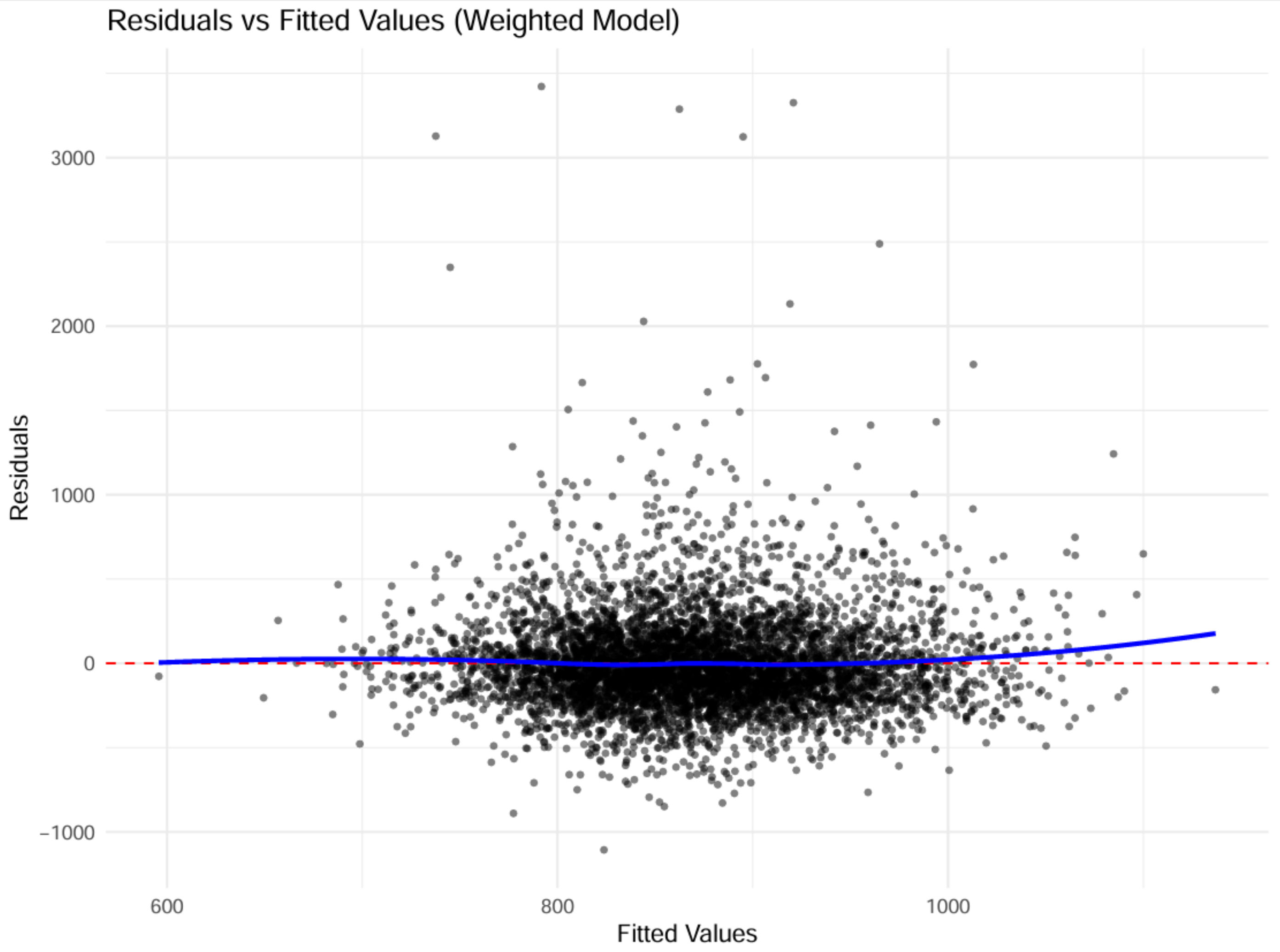

Figure S13 Homoscedasticity of residuals. The scatter plot of residuals and fitted values shows no obvious trend, indicating homogeneity of variance.

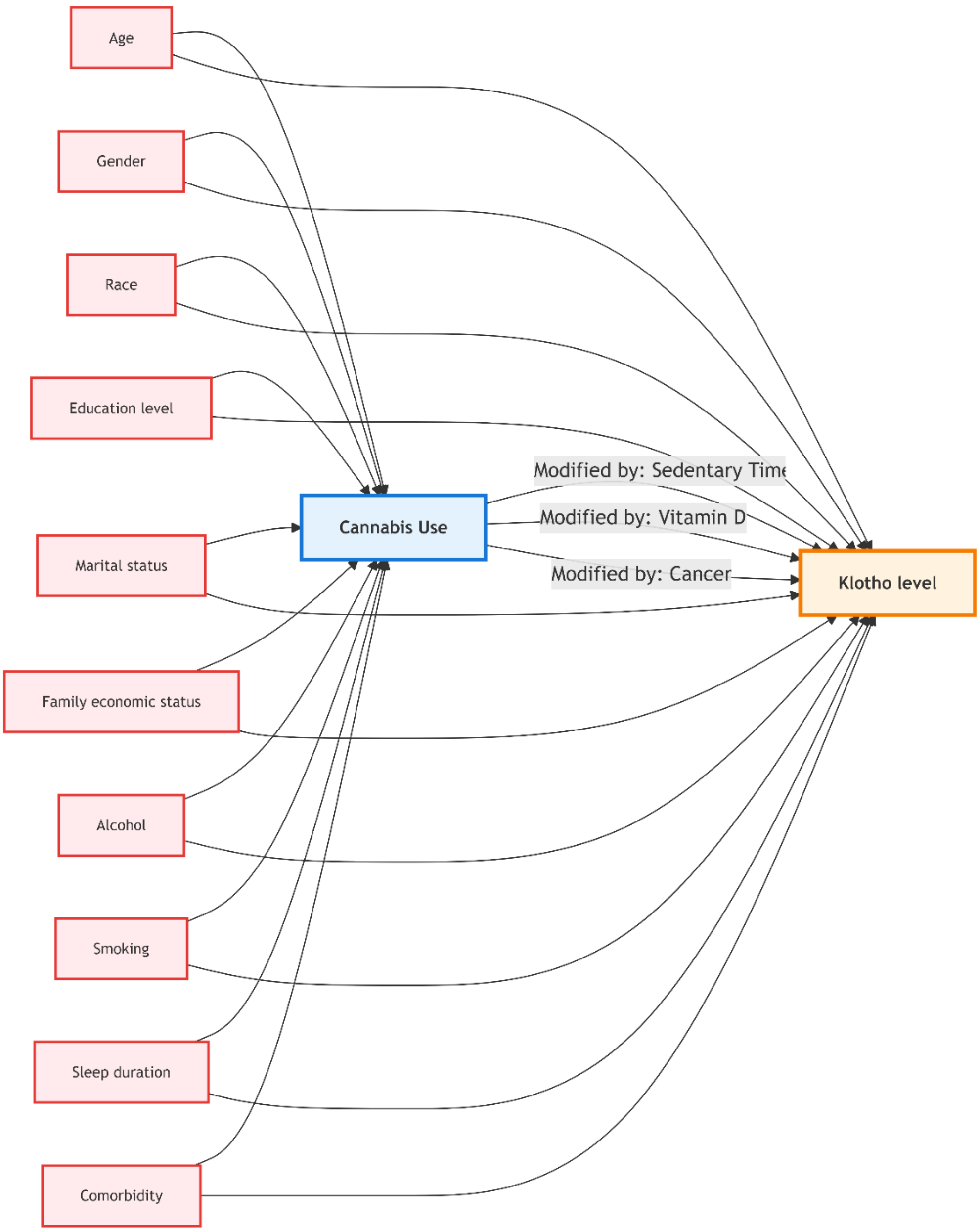

Figure S14 Directed Acyclic Graph (DAG) illustrated the assumed causal relationships between cannabis use (exposure), serum Klotho levels (outcome), potential confounders, and effect modifiers. Confounders (age, gender, race, education level, marital status, family economic status, alcohol consumption, smoking, sleep duration, comorbidity) were hypothesized to influence both cannabis use and Klotho levels. The association between cannabis use and Klotho levels was modified by sedentary time, renal function (eGFR), vitamin D, and cancer (identified via statistically significant interaction analyses). Note: This DAG represented theoretical assumptions, not empirically confirmed causal relationships.
