# Supplementary material for: Association between cannabis consumption and serum Klotho levels in middle-aged U.S. adults: NHANES cross-sectional analysis
Source: J Cannabis Res. 2026 Jan 6;8:18. doi: 10.1186/s42238-025-00380-x (PMC12870139; doi:10.1186/s42238-025-00380-x)
Supplement: Supplementary file 2 — Supplementary Material 2. [file 42238_2025_380_MOESM2_ESM.pdf]

Supplementary Table S1 Normality test for continuous variables

**Continuous variables**	**Kolmogorov-Smirnov Test (P value)**	**Skewness**	**Kurtosis**
Age	<0.001	0.05	1.84
PIR	<0.001	0.12	1.5
Sleep duration	<0.001	-0.14	3.96
Sedentary activity	<0.001	0.62	2.88
WWI	<0.001	0.21	3.11
Age started smoking cigarettes regularly	<0.001	0.72	7.78
Age when first tried cannabis	<0.001	2.37	12.4
Last time used cannabis	<0.001	7.25	130
eGFR	<0.001	-0.88	4.95
Vitamin D	<0.001	0.87	5.28
Klotho	<0.001	2.13	15.9

Abbreviations: eGFR, estimated glomerular filtration rate; PIR, family income-to-poverty ratio; WWI, Weight-adjusted waist circumference index.

Supplementary Table S2 Univariate analysis of the associations between serum Klotho concentration and baseline variables.

**Variables**	**Estimate, (95%CI)**	***P*-value**
Gender		
Female	Ref	
Male	-38.01 (-57.54, -18.66)	<0.001
Age	-1.07 (-2.77, 0.63)	0.215
Race		
Mexican American	Ref	
Other Hispanic	15.20 (-19.65, 50.04)	0.388
Non-Hispanic White	-14.04 (-40.81, 12.74)	0.300
Non-Hispanic Black	67.73 (29.98, 105.49)	<0.001
Other Race	14.15 (-31.51, 59.80)	0.539
Education level		
Less than 9th grade	Ref	
9–11th Grade	12.66 (-23.62, 48.94)	0.489
High school graduate/ GED or equivalent	-2.67 (-34.19, 28.86)	0.867
Some college or AA degree	22.88 (-11.45, 57.20)	0.188
College Graduate or above	40.49 (7.19, 73.79)	0.018
Marital status		
Married	Ref	
Widowed	30.14 (-36.52, 96.81)	0.371

Divorced	10.52 (-17.89, 38.94)	0.463
Separated	-9.28 (-45.73, 27.18)	0.614
Never married	23.68 (-20.01, 67.36)	0.284
Living with partner	-25.85 (-71.26, 19.57)	0.260
PIR	1.68 (-5.07, 8.44)	0.621
WWI	-17.26(-30.79, -3.74)	0.013
Sleep duration (hours)	-14.29 (-20.94, -7.63)	<0.001
Sedentary activity (minutes)	0.0008 (-0.04, 0.04)	0.971
Alcohol consumption		
<12drinks/year	Ref	
≥12drinks/year	-67.55 (-89.98, -45.11)	<0.001
Age started smoking cigarettes regularly	-1.76 (-4.14, 0.63)	0.146
Age when first tried cannabis	0.18 (-2.43, 2.79)	0.893
Last time used cannabis (days)	0.65 (-0.17, 1.47)	0.117
Hypertension		
No	Ref	
Yes	-14.85 (-33.64, 3.94)	0.120
Diabetes		
No	Ref	
Borderline	-30.16 (-76.73, 16.41)	0.201
Yes	12.47 (-15.30, 40.24)	0.374
CVD		
No	Ref	
Yes	-28.52 (-70.03, 12.30)	0.175
COPD		
No	Ref	
Yes	-12.4 (-53.64, 28.85)	0.551
Stroke		
No	Ref	
Yes	-73.10 (-132.45, -13.75)	0.016
Cancer		
No	Ref	
Yes	-10.42 (-40.14, 19.30)	0.487
eGFR	1.49 (0.95, 2.04)	<0.001
Vitamin D	-0.23 (-0.63, 0.17)	0.263

Note：Univariate analysis were conducted by the weighted generalized linear regression method.

Abbreviations: CI: confidence interval; COPD, chronic obstructive pulmonary disease; CVD, cardiovascular disease; eGFR, estimated glomerular filtration rate; PIR, family income-to-poverty ratio; WWI, Weight-adjusted waist circumference index.

Supplementary Table S3 Collinearity screening between cannabis use and other variables.

Variables	Variance inflation factor
Gender	1.16
Age	1.30
Race	1.09
Education level	1.32
Marital status	1.11
PIR	1.42
WWI	1.29
Sleep duration	1.04
Sedentary activity	1.10
Alcohol consumption	1.07
Smoking status	1.21
Age started smoking cigarettes regularly	1.09
Cannabis use	1.15
Cannabis use frequency	1.09
Age when first tried cannabis	1.03
Hypertension	1.17
Diabetes	1.17
CVD	1.14
COPD	1.08
Stroke	1.08
Cancer	1.04
eGFR	1.15
Vitamin D	1.11

Note-1: Variance inflation factor = $1/(1-R^2)$.

Note-2: The variables with variance inflation factor >5 will be regarded as collinear variables and cannot be included in the multiple regression model.
